# Protein function prediction using domain families

**DOI:** 10.1186/1471-2105-14-S3-S5

**Published:** 2013-02-28

**Authors:** Robert Rentzsch, Christine A Orengo

**Affiliations:** 1 Robert Koch Institut, Research Group Bioinformatics Ng4, Nordufer 20, 13353 Berlin, Germany; 2 Institute of Structural and Molecular Biology, University College London, Darwin Building, Gower Street, London WC1E 6BT, UK

## Abstract

Here we assessed the use of domain families for predicting the functions of whole proteins. These 'functional families' (FunFams) were derived using a protocol that combines sequence clustering with supervised cluster evaluation, relying on available high-quality Gene Ontology (GO) annotation data in the latter step. In essence, the protocol groups domain sequences belonging to the same superfamily into families based on the GO annotations of their parent proteins. An initial test based on enzyme sequences confirmed that the FunFams resemble enzyme (domain) families much better than do families produced by sequence clustering alone. For the CAFA 2011 experiment, we further associated the FunFams with GO terms probabilistically. All target proteins were first submitted to domain superfamily assignment, followed by FunFam assignment and, eventually, function assignment. The latter included an integration step for multi-domain target proteins. The CAFA results put our domain-based approach among the top ten of 31 competing groups and 56 prediction methods, confirming that it outperforms simple pairwise whole-protein sequence comparisons.

## Background

### Motivation

Most proteins consist of multiple domains [1,2], owing to the modular recombination of domains throughout protein evolution ('domain shuffling') [3,4]. This means that there exist many more pairwise homology relationships between individual domain sequences than between whole-protein sequences. Exploiting this fact can increase the sensitivity of protein function prediction (ProFP) approaches, as has already been demonstrated for a simple protocol based on pairwise sequence comparisons in [5]. Extending on this, it should be possible to improve any ProFP method that is based on protein sequence comparisons by performing these comparisons on the domain level instead and, subsequently, integrating the results obtained for all domains. One of the key challenges in this is the association of domain families with protein functions.

### Existing methods

There exist two (partly) manually curated meta-resources that aim to functionally characterise protein domain families, InterPro [6,7] and CDD [8]. However, most of this information is still stored in free text. While the InterPro2GO mapping [6,7], including Pfam2GO, is manually curated, several methods to derive domain-specific annotations automatically have been published to date. They all use protein GO annotations and domain assignments, the latter made using sets of predefined domain families such as those found in Pfam [9].

Schug and colleagues developed a rule-based approach [10]. A set of GO-annotated proteins is initially scanned against a set of domain families using (RPS-)BLAST [11]. For a given family, the hit list is first sorted by p-value. Different types of rules are then associated with specific p-value thresholds. For example, a 'single function' rule is generated when the first N hits in the list have only a single, shared most specific GO annotation. Other rule types are more sophisticated, taking into account varying annotation specificity and missing annotations.

The GOTrees method [12] made it possible to also resolve joint functions of consistently co-occurring domains. It initially models the domain content of all training proteins as a presence/absence vector. The domain vectors are then mapped to GO terms, creating a decision tree for each term that best separates those proteins (i.e., single domains or domain combinations) that are assigned the term from the rest.

Two protocols that extend on the Pfam2GO approach were later presented by Forslund and Sonnhammer [13]. MultiPfam2GO is a straightforward generalisation to multi-domain sets: the smallest possible set that consistently occurs in proteins associated with a given GO term (set) is associated with that term (set). The second protocol accounts for sparse or missing GO annotations and domain assignments, using a naïve Bayesian network classifier to associate domain sets with GO terms (or term sets) probabilistically.

SCOP2GO [14] associates SCOP structural domains (folds) with GO terms. The domain composition of all proteins assigned a given term T is collected in a matrix, followed by an iterative protocol. First, the domain D with the highest occurrence count in the matrix is associated with T, labelling all instances of D in protein sets as T-associated. All co-occurring domains are labelled as not T-associated. Second, all D-containing proteins are removed. The protocol iterates until no protein is left. For each domain D, the likelihood of it 'encoding' function T is then calculated. In this manner, functions can be associated with multiple domain types, and vice versa.

### Overall strategy

**Figure 1:**
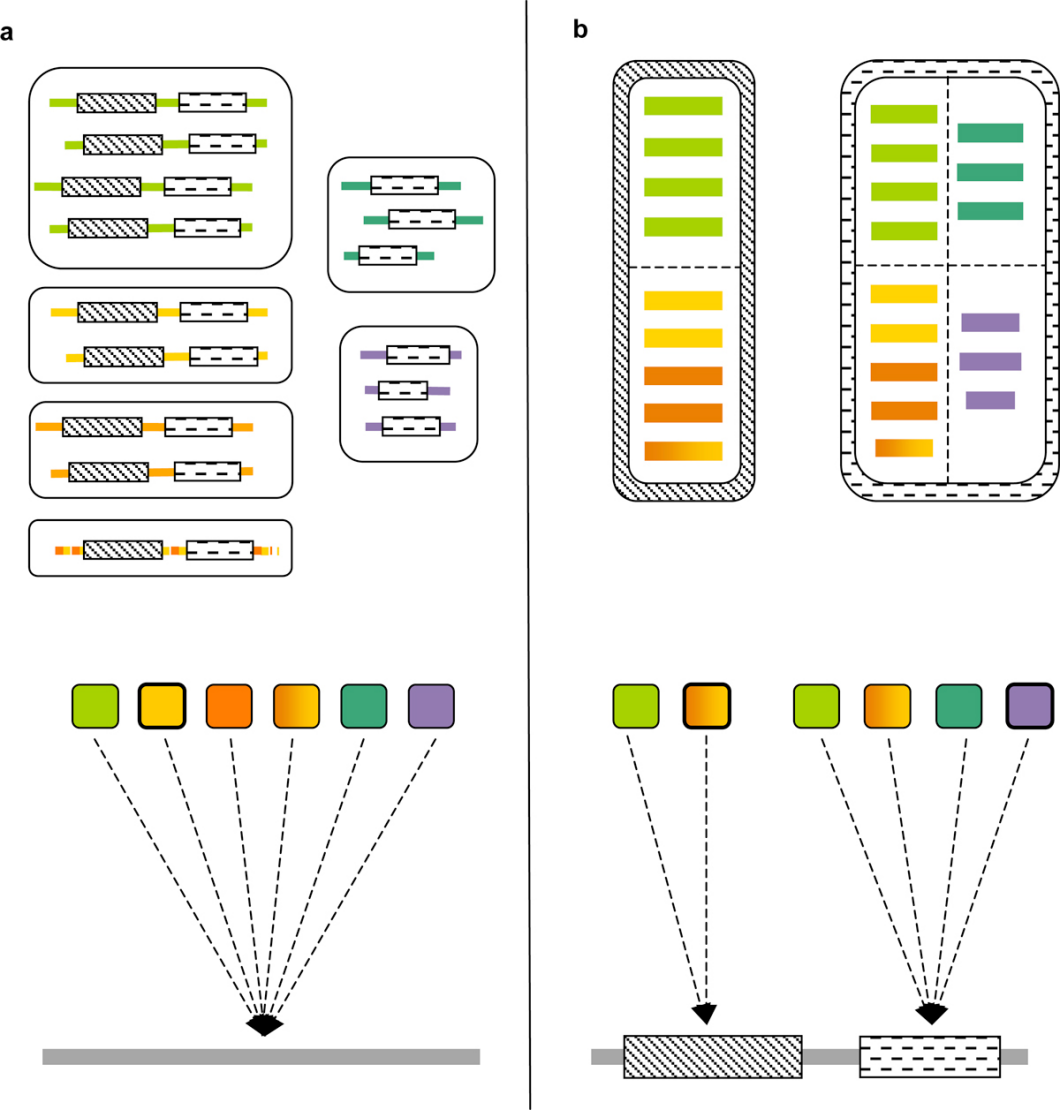
**Protein families versus protein domain families in protein function prediction**. (**a**) Six evolutionarily related families of proteins with assigned domains. The proteins are coloured by function, where mixed colouring indicates multi-functionality. The domain dashing patterns indicate different superfamilies. A protein family resource would build one model per protein family (middle; coloured squares) and scan target proteins with these models, to assign them to families. (**b**) The domains from the proteins in (a) in their domain superfamilies, coloured by the function of the respective parent protein. Each superfamily is subdivided into functional families (dashed lines), based on the protocol described in the main text. Note that domains from functionally very similar proteins (red, orange, yellow) can go to the same family. The domain-based protein function prediction protocol first identifies domains in the target protein (bottom) and then scans each domain sequence with the functional family models available for its domain superfamily (middle). Each functional family is associated probabilistically with different whole-protein functions. Based on the family assignments of the individual domains, a combined function prediction for the whole protein is made. The best-scoring protein and domain family models are highlighted with a bold border in (a) and (b), respectively.

A common sequence-based ProFP approach is the assignment of target proteins to an existing library of protein families, for example, those found in PANTHER [15] or TIGRFAMs [16], where each family is associated with an overall protein function (Figure 1a). Correspondingly, our method assigns each identified domain in a given target protein to an initially established library of domain families (Figure 1b), where each family is associated probabilistically with a set of protein functions. In this manner, we take into account the appearance of domains in different multi-domain and, therefore, functional contexts. The function assignments obtained for all domains are further integrated into whole-protein function predictions using a simple protocol.

Importantly, while the already existing domain superfamilies as defined in CATH [17] and populated in Gene3D [18] collect structurally (but not necessarily functionally) similar domain sequences, they provided the framework for family identification: each superfamily was divided into families.

### Comparison with GeMMA

We have already described a protocol for family identification below the superfamily level, GeMMA [19]. This was based on a high-throughput method for profile-based sequence clustering (sampling step). Families were identified based on 'cutting' the produced dendrogram of sequence clusters at a fixed level of clustering granularity (selection step). The granularity setting was derived by training on a small set of hand-curated domain superfamilies.

The 'functional domain families' (FunFams) that we used to predict protein function in CAFA 2011 were derived using the same sampling method, but with a new, supervised selection procedure. This directly uses Gene Ontology (GO) [20] protein function annotation data (similar to the protocols described above), and can therefore partition the superfamily dendrogram into functional families much more precisely. The new strategy further makes it possible to cluster the superfamilies non-exhaustively in the sampling step, that is, to only cluster those member sequences that are functionally characterised to some extent.

**Figure 2:**
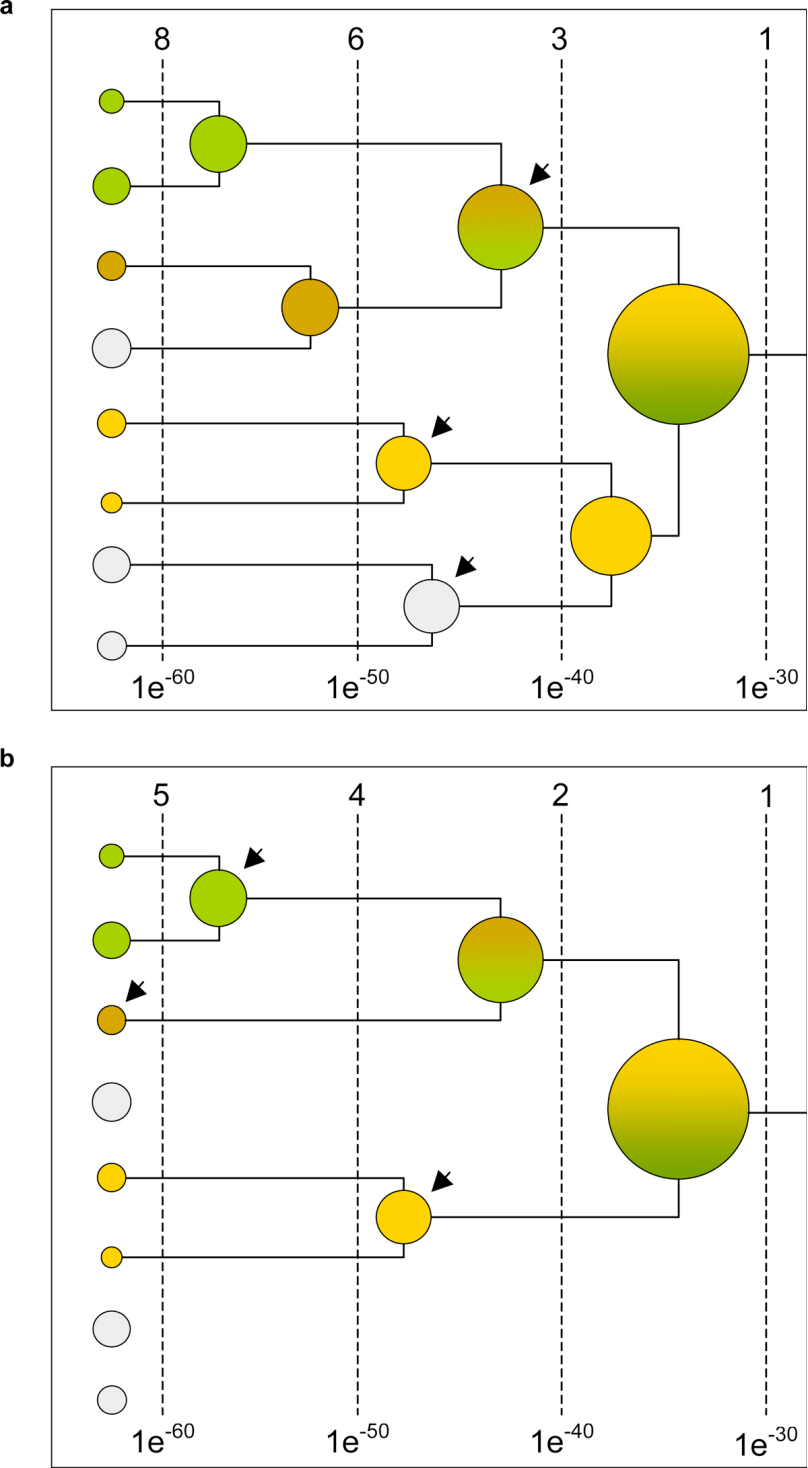
**Comparison of the GeMMA and FunFam family identification protocols**. (**a**) The partial GeMMA clustering dendrogram of a sequence superfamily. Different colours correspond to the protein functions associated with the clusters; grey indicates a lack of annotations. (**b**) The corresponding part of the dendrogram when using the FunFam protocol. Note that unannotated starting clusters (grey) are here removed prior to clustering. The COMPASS [33] E-values at the bottom of both subfigures reflect the maximum sequence profile similarity observed between any two clusters at a given point, which decreases in the course of clustering [19]. The number of clusters (shown in this part of the dendrogram) that still exist when stopping the clustering at a given granularity level is stated at the top. Arrows indicate which clusters are eventually selected to represent functional families.

Figure 2 summarises the differences between the original GeMMA protocol (a) and the CAFA FunFam protocol (b) by use of an example. Both parts shows the partial clustering dendrogram of a given domain superfamily, processed with either method. The clusters are coloured by the functions (annotations) of the sequences they contain, respectively; grey indicates a lack of annotations.

An immediately obvious difference between the two parts of Figure 2 is that the grey clusters are not part of the dendrogram in part b. This is because these clusters represent (parents of) starting clusters that do not contain annotated sequences (see below for how the starting clusters are derived and annotation quality requirements). Unannotated starting clusters are removed prior to clustering in the FunFam protocol.

The GeMMA protocol stops the clustering at a fixed granularity level of E = 10^-40^. In the example in Figure 2 this would produce three families for the shown part of the superfamily dendrogram (a; arrows). In contrast, the FunFam protocol identifies different clusters as putative families (b; arrows). This is based on tracing the dendrogram as a whole and identifying the individual points at which clusters (sequences) with different associated functions get merged.

## Methods

### Domain family identification

This section describes the family identification protocol step-by-step, for an individual Gene3D domain superfamily.

#### Step 1 - Data preparation

All domain sequences in the superfamily are pooled into a single FASTA file. The UniProtKB-GOA [21] annotations for all the corresponding parent proteins are obtained and filtered for high-quality annotations. In this, the following annotations are kept: all non-IEA (whether manually derived or curated) GO annotations to proteins in SwissProt and TrEMBL [22] as well as all IEA annotations to proteins in SwissProt that were made using either the SwissProt Keyword2GO [23] or the EC2GO [24] mapping methods. Both the latter methods have been shown to exhibit higher accuracy than the remaining ones [23,25] and, owing to the restriction to SwissProt proteins, primarily represent a 'translation' of manually curated SwissProt keyword and EC [26] annotations to GO annotations. Finally, only the most specific terms are kept for each protein, that is, any GO parent terms are removed.

The datasets used were Gene3D v9.2, GO term definitions dated 15/05/2010 and UniProtKB-GOA annotations dated 24/09/2010.

#### Step 2 - Sequence clustering

The superfamily sequences are pre-clustered at 90% pairwise sequence identity using the high-speed but low-precision clustering tool CD-HIT [27]. All non-annotated clusters (such that do not contain at least one sequence with high-quality GO annotation(s); see above) are removed from the set of obtained non-redundant clusters. This results in a set of 'starting clusters' for the profile-based hierarchical clustering algorithm described in [19], which is now used to further cluster the starting clusters until only a single cluster remains.

#### Step 3 - Cluster analysis

**Figure 3:**
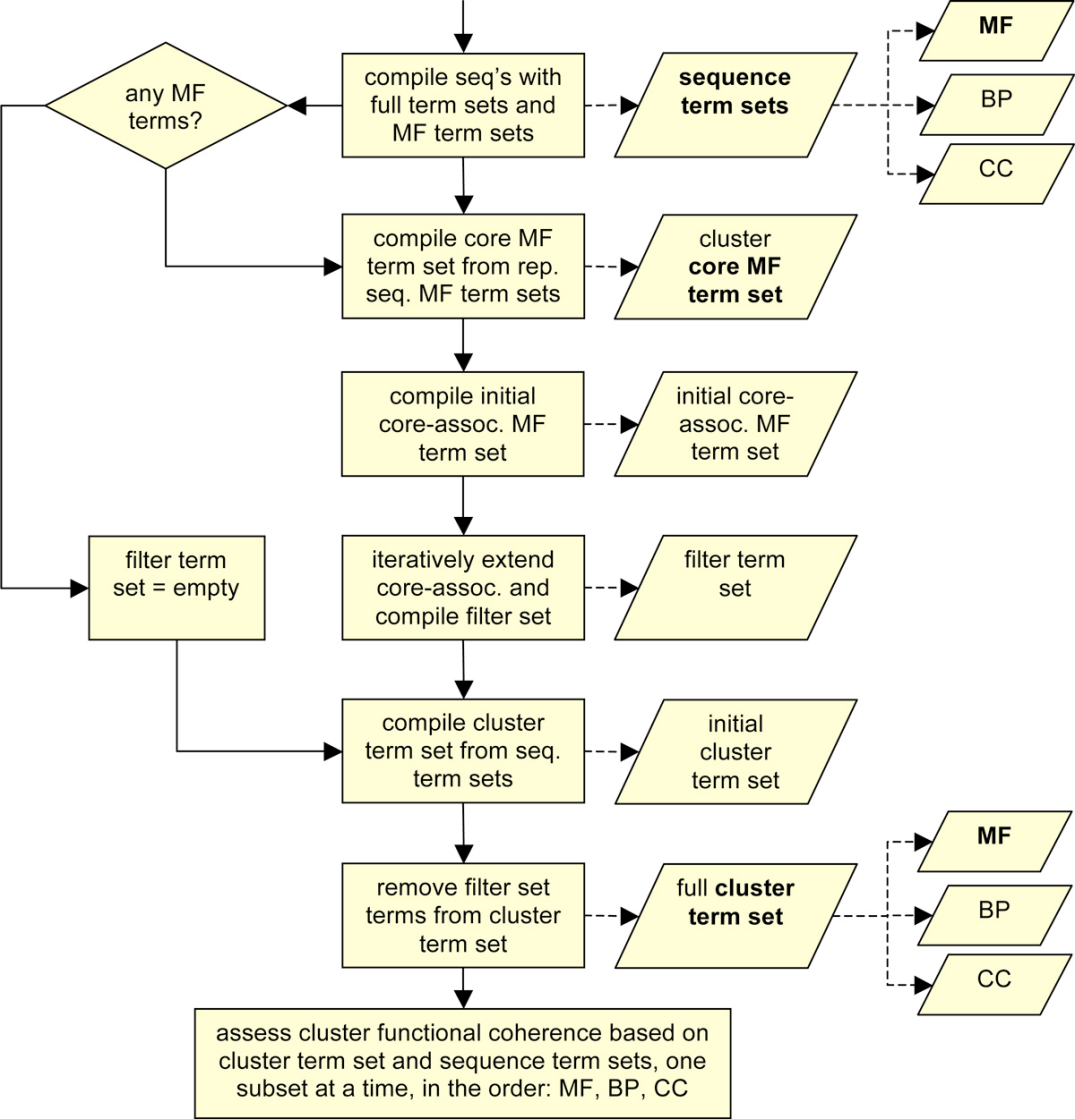
**The sequence and cluster term sets involved in assessing cluster functional coherence**. The sequences in a given domain sequence cluster are associated with different GO term sets via their parent proteins. Each sequence term set can be split into MF, BP and CC term subsets. If any of the sequence MF term sets is not empty, a set of core MF terms for the cluster is compiled from all sequence MF term sets, and, via further, intermediate steps, a set of terms to be ignored in cluster assessment is compiled. If none of the sequence term sets contains MF terms, the filter term set is an empty set. In the next step, the initial cluster term set is prepared as the union of all representative sequence term sets. From this set, any terms that are also found in the filter term set are removed (filtered), yielding the final cluster term set. Like the sequence term sets, the cluster term set can be split into MF, BP and CC subsets. The term-type specific sets of the representative sequences are compared with those of the cluster as a whole, respectively, to assess the functional coherence of the sequences in the cluster. Key term sets in the described process are highlighted in bold.

All sequence clusters in the generated dendrogram are processed and assessed for functional coherence using the following assumptions and algorithms. The assessment protocol was designed according to the requirement of using GO annotation data; note that it could be much simpler for EC data, for example, as will become clear in the following. Figure 3 serves as a guideline through the cluster analysis workflow, also listing the most important term sets used in the process.

#### The sequence and cluster term sets

The parent proteins of the domain sequences in a given cluster are associated with high-quality GO annotations (see above), the *sequence term sets*. The union of all sequence term sets is the *cluster term set*. Terms of the molecular function (MF) type define subsets in both cases: the *sequence MF term sets *and the *cluster MF term set*. Due to the nature and character of GO annotations, the sizes of all sequence-specific term sets and the specificity of the terms they contain vary.

#### The cluster core term set

While a detailed discussion of the relationship between GO MF annotations and individual protein domains (and the relationship between whole-protein and domain function in general) is beyond the scope of this work, the following simple assumptions should hold. First, the more domains a protein has the more functionally complex it will usually be, and the more MF terms it will have (and vice versa). Therefore, small *sequence MF term sets *(particularly of size 1) associated with domains in a cluster often stem from single-domain proteins, capturing only the function of the particular domain under inspection. Second, essential terms that describe key parts of protein function (e.g., a certain enzymatic activity) are less likely to be missing from a protein's MF annotations than less important, 'ancillary' terms (e.g., a cofactor-binding activity that is already implied in the catalytic activity). This is because such partial functions are less likely to be explicitly confirmed and/or reported by authors in the first place, and, therefore, less likely to be curated and/or annotated later on. Taken together, the *sequence MF term sets *of minimum size are a good starting point for compiling a *core term set *('core set') for a cluster, based on which its functional coherence is subsequently assessed.

**Figure 4:**
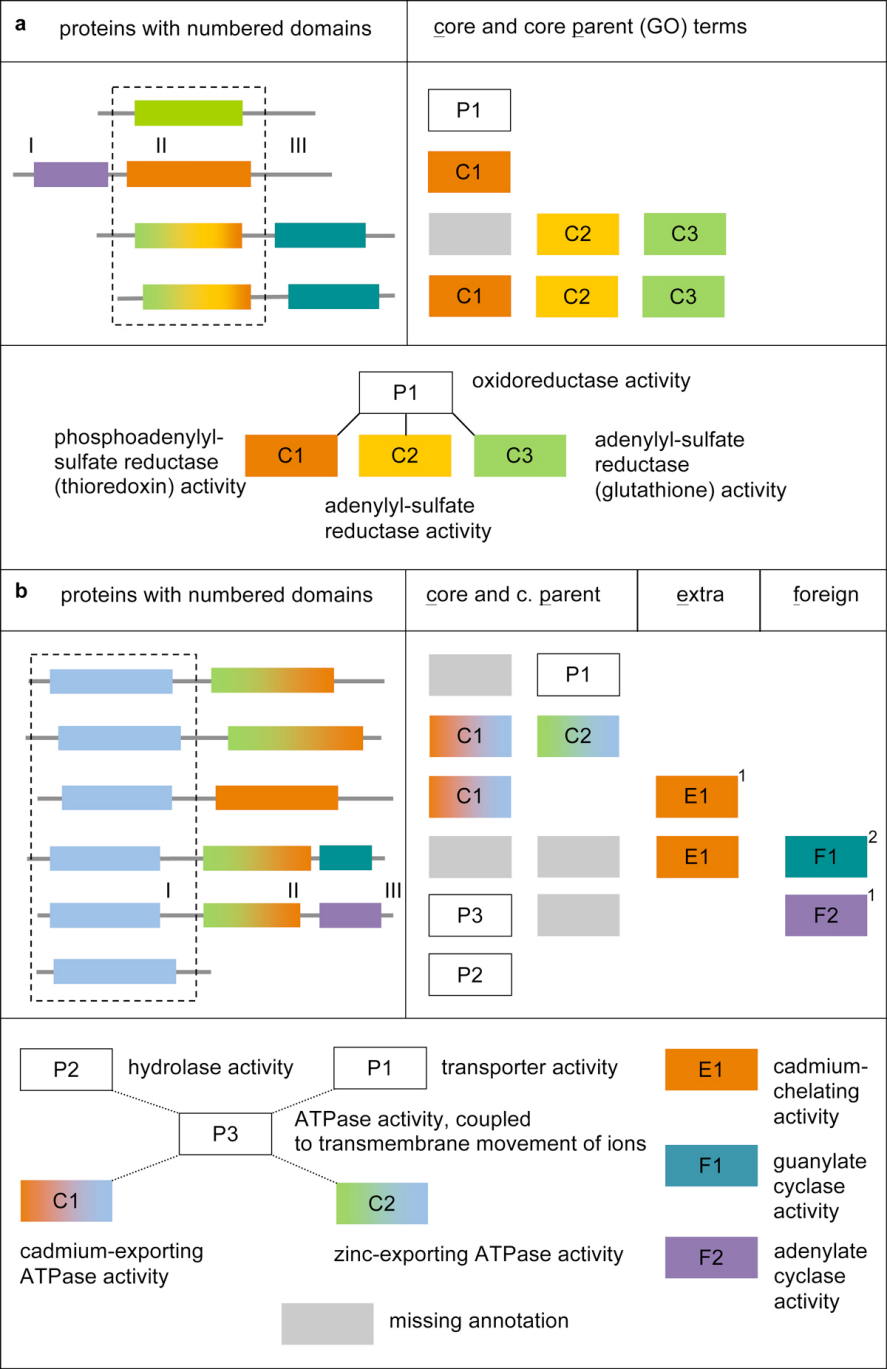
**Two example domain sequence clusters and the associated protein function annotations**. All domains are coloured and labelled according to their true functions; the available high-quality GO annotations of the parent proteins are shown on the right, respectively. The GO terms are coloured according to the protein functions they describe, and their hierarchical relationships in the GO DAG are shown at the bottom, respectively (dashed lines represent omitted intermediate terms). Both clusters (dashed boxes) represent functional domain families according to criteria outlined in the main text. The three reductase functions in (**a**) are closely related, as indicated by the three-functional cluster member sequences. In (**b**), the hydrolase function is perfectly conserved among all member domain sequences. Note that both the true domain functions and the types of the available annotations (core, extra and foreign-domain; see main text) are 'invisible' to the core set identification protocol. The small numbers next to specific functions indicate in which step of the iterative protocol described in the main text they are identified as 'core-associated' functions (see annotation editing section).

Figure 4a shows a simple example annotation scenario, where a cluster contains four domain sequences with conserved reductase activity; these are the centred domains (domain II) in the parent protein chains on the left, respectively. This domain is multi-functional in the sense that it can perform the same reaction on a range of highly similar (co-)substrates (Figure 4a; bottom). For simplicity, the other domains in these proteins are assumed to have scaffold function only. The (partially incomplete) annotations of the parent proteins are shown on the right. In this example the initial core set is (C1, P1), based on the two sequences associated with a single MF term (the smallest occurring sequence MF term sets).

After compiling the initial core set as described above, this is processed further, in two steps. First, for any term occurring with those sequences whose MF term sets have a size greater than the minimum size observed (those that were not considered when compiling the initial core set; see above), the presence of GO parent terms in the initial core set is assessed. All terms that are (more specific) children of core terms and are not already part of the initial core set are added to this set. In the example in Figure 4a, these are C2 and C3, both children of P1. Second, any parent terms in the (now extended) initial core set are removed (P1). The resulting, final core set comprises the most specific out of all putatively essential annotations that occur in a cluster (C1, C2 and C3 in this case), and only those.

In addition to the simple example scenario in Figure 4a, as discussed above, part b shows a more complex situation. Here, a range of essential (core), non-essential (extra) and foreign-domain annotations is associated with the domain sequences in the inspected cluster (domain I in the parent protein chains on the left, respectively), via their parent proteins. The core set is established according to the above-described steps. Therefore, the initial core set is (P1, P2) and the final core set is (C1, C2).

#### Annotation editing

The core term set as introduced above is a heuristic means to identify and remove non-essential and foreign-domain annotations (see above) from the cluster term set. This editing process is important for a sensible assessment of functional coherence, as described further below. First, two term sets are created. The core terms and all their GO parent terms together form the initial *core-associated *term set. The *filter *set is initially empty. It is populated in the following iterative procedure and subsequently used to filter the cluster term set.

In the first iteration, all sequence MF term sets with greater than minimum size (see above) are analysed one by one, as follows. First, all terms in the set are checked for whether or not they are core-associated. If at least one such term is found, all terms in the set that are not yet registered as core-associated are denoted. After assessing all sequence MF term sets, it is checked whether any novel core-associated terms have been identified. If this is the case, these terms are added to the core-associated term set, together with the union of all their GO child and parent terms. All these terms are also added to the filter set. Subsequently, all sequence MF term sets are assessed afresh. The iterative term set assessment procedure continues until no further core-associated terms are identified. As a result, the filter set contains all core-associated terms identified (including transitive identification), except the core terms themselves.

The core-associated terms that are added to the filter set in the process described above are expected to represent non-essential and foreign-domain annotations, with respect to the function(s) of the domain sequences in the processed cluster. The 'associated' here means that these terms co-occur with core terms, whilst not being core terms themselves.

The example in Figure 4b can serve to illustrate how the filter term set is progressively populated with non-essential and foreign-domain terms. The core set here is (C1, C2). In the first iteration of assessing the sequence MF term sets, E1, a non-essential (extra) term, is identified as core-associated (iteration indicated by the small number next to the term). This is based on its co-annotation with C1, a core term. At the same time, F2, a foreign-domain term, is identified as core-associated too, based on its co-annotation with the core parent term P3. F1, however, is only identified as core-associated in the second iteration, due to its co-annotation with E1, a core-associated term. In the simpler scenario in Figure 4a no extra or foreign-domain terms are annotated. Therefore, no core-associated terms are identified in the single iteration that is carried out.

#### Coherence assessment

After core set identification and, based on this, editing the cluster term set, the functional coherence of the cluster is assessed. The assessment is based on the most suitable available GO term type, in the following order: molecular function (MF), biological process (BP) and cellular component (CC). This corresponds to the relevance of each term type when trying to identify functionally coherent sequence families. Only if the term set of a given cluster does not contain terms of a specific type at all, the next type in the above list is used. Note that only MF annotations directly describe the mechanistic function(s) of individual proteins (and domains), and that the above-described steps only concern the MF annotations, leaving the BP and CC parts of the cluster term set untouched.

After determining which GO term type is used to assess the functional coherence of the cluster, the assessment term type T_a_, the protocol proceeds with compiling the necessary data. First, all T_a_-type terms are collected from the cluster term set, forming the cluster T_a _term set. Second, all sequences with at least one T_a_-type term are compiled, and the corresponding sequence T_a _term sets determined. The following step marks the core of the assessment protocol. All sequence T_a _term sets are compared with the cluster T_a _term set. If at least one of the sequence term sets covers all the terms in the cluster term set, the cluster is judged functionally coherent. The overall strategy outlined is tolerant towards inconsistent and missing GO annotations, unlike, for example, a simple rule requiring all sequence annotations for a given cluster to match exactly.

#### Step 4 - Dendrogram pruning

Finally, after assessing all domain sequence clusters as described above, those that are not judged functionally coherent are removed from the clustering dendrogram. This splits the dendrogram into sub-trees, since the level of cluster functional coherence generally decreases between the leaf nodes and the root node. Only the root clusters of all derived sub-trees are retained, forming a set of functional families in the superfamily.

### Protein function prediction

This section describes how the identified domain families were used for protein function prediction in CAFA 2011.

#### Step 1 - Generating family model libraries

For each family identified in a given superfamily an alignment is generated using MAFFT with high-quality settings ('--amino --localpair --maxiterate 1000') and, based on this, a profile HMMs is built using the HMMER3 [28]*hmm_build *command with default settings.

#### Step 2 - Associating families with functions

Each family is further associated probabilistically with protein functions, in the following manner. First, for each most specific GO term that is associated with the family (via one or more of the corresponding parent proteins), it is counted how often it is associated with individual sequences. The resulting number *T *ranges from 1 to the number of domains in the family *N*. Second, all counts are up-propagated in the GO DAG so that coarser terms can be supported by more specific child terms. Finally, the family is associated with all terms that received a count in the above process, each with a term-specific probability value *p*. This is simply derived as the normalised term occurrence count or annotation frequency: *p *= *T*/*N*, ranging from 0 to 1. Note that this simple approach is agnostic about the overall domain architecture of the parent proteins and thereby implicitly takes into account the likelihood of other domains (with other functions) to co-occur with domains from the processed family in known proteins.

Figure 1b at the start of this paper can serve as a worked example for the above-described process. Here, each colour corresponds to a specific GO term. For example, the 'yellow-orange' family in the left superfamily would have the following functions associated: 'yellow', with probability 3/6 = 0.5; 'orange', with probability 3/6 = 0.5. This is assuming that a yellow and orange colouring of the same sequence means it is associated with both functions (e.g., using two different enzyme substrates) via its parent protein (Figure 1a). Accordingly, the violet family in the right superfamily in Figure 1b has only the function 'violet' associated, with probability 3/3 = 1.0.

#### Step 3 - Family and function assignment

All target proteins are first assigned CATH domains, using the standard protocol that is also followed for generating the Gene3D resource [29]. The identified domain sequences in each superfamily are then scanned against the superfamily's family model library using the HMMER3 *hmm_scan *command with default options. Based on the results of this, the sequences are associated with GO terms and corresponding p-values (see above). For CAFA 2011, the simplest possible term assignment protocol led to the best observed performance: only the top-scoring (bit score) model is taken into account and all GO term probabilities associated with the corresponding family are assigned to the query sequence. Finally, a simple integration procedure provides the whole-protein GO term assignments: the whole-protein probability score for each GO term is set to the maximum domain-based probability score obtained for the term, respectively.

The target protein at the bottom of Figure 1 has domains in the 'yellow-orange' and 'violet' families (Figure 1b) that have already served as an example in the previous section (the best-hit models are highlighted with a bold border in Figure 1). According to the process described above and the term probabilities associated with these families (see previous section), its predicted annotation would therefore be: 'orange' (0.5); 'yellow' (0.5); 'violet' (1.0).

A more sophisticated approach that uses model-specific thresholds when scanning the domain sequences, takes into account hits to all models hit above the respective threshold and, based on relative model weights (derived from the absolute hit scores), integrates the term probabilities over all models, did not lead to superior performance.

### Measuring performance

#### Family quality

For the enzyme superfamily benchmark, 467 Gene3D superfamilies with EC annotations were compiled. A domain superfamily belonged to this set if it had at least two different four-digit EC annotations (EC4s) associated with its domains' parent proteins. EC annotations were taken from the UniProtKB-GOA file, where they are stated for EC2GO-mapped terms. Retrieving the ECs from UniProtKB directly, with a query that filters for high-quality annotations only ["not (name:putative or name:probable or name:potential or name:possible or name:likely or name:unknown or name:uncharacterised or name:uncharacterized) and fragment:no and precursor:no and reviewed:yes and EC:*"], did not alter the results markedly.

A simple EC-based performance measure for arbitrary family partitionings was used, which equally considers the average number of different EC4s per family (diversity, a specificity measure) and the average number of families per EC4 (division, a sensitivity measure). The raw performance score is thus given as the arithmetic mean of average family diversity and division. The lowest value it can assume is 1, that is, if none of the produced families are functionally diverse and there is only one family per function. Averaging over all analysed superfamilies yields an average performance (AP) score. The degree to which the underlying raw scores vary reflects the degree to which the quality of the produced family partitionings varies for the different superfamilies processed.

#### Protein function prediction performance

The CAFA 2011 assessment methodology is described elsewhere in this special issue.

## Results

### Family identification in Gene3D superfamilies

Using the described protocol, families could be identified in 1,793 (~75%) of the 2,382 protein domain superfamilies in Gene3D 9.2. For the remaining ~25% of (mostly small) superfamilies, no high-quality GO annotation data was available. The union of GO annotations used was associated with 338,116 distinct UniProtKB protein sequences.

### Family quality

**Figure 5:**
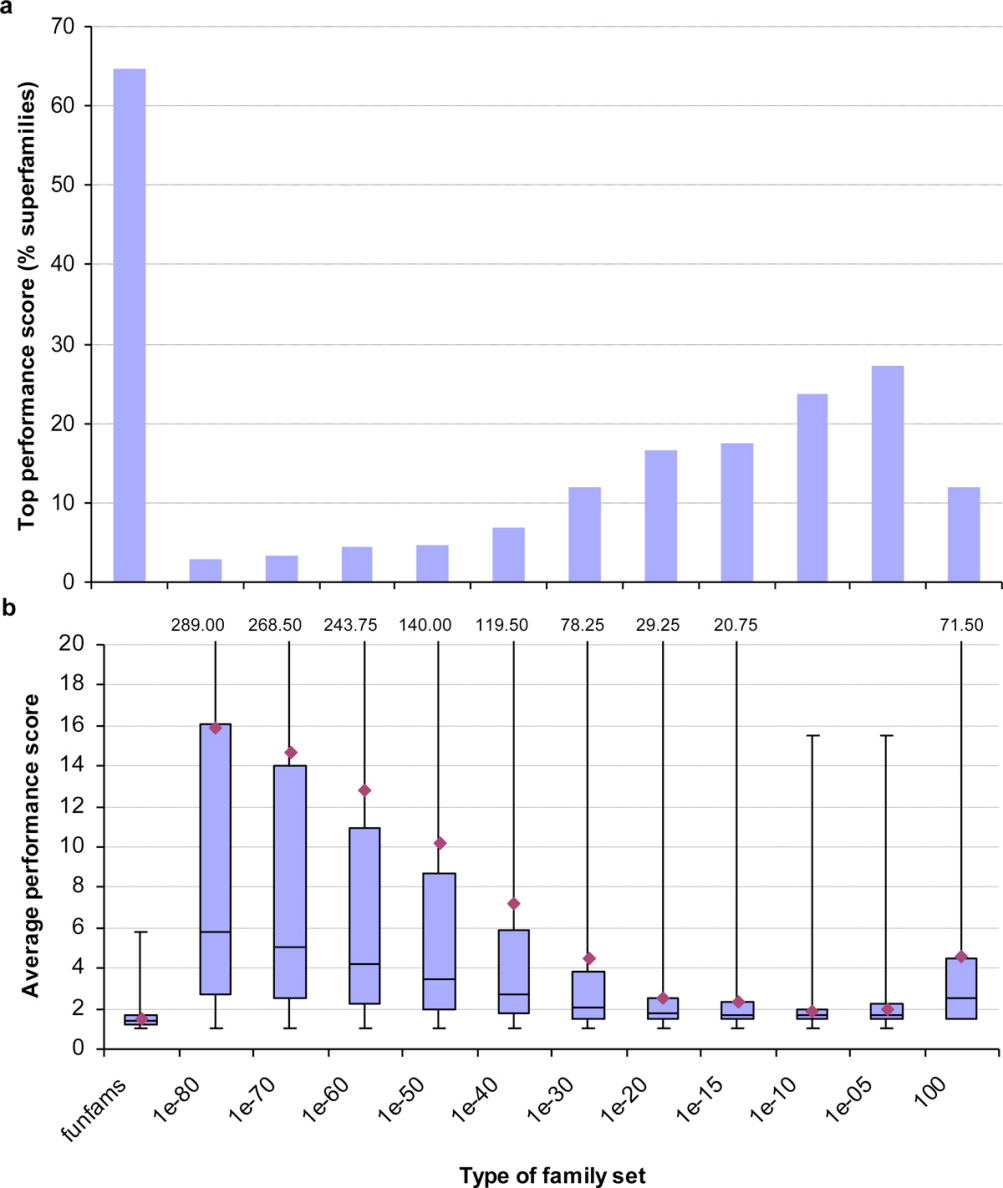
**The relative quality of the FunFam and different fixed-granularity GeMMA family partitionings obtained for 466 enzyme domain superfamilies in Gene3D**. (**a**) The fraction of superfamilies for which a given method or clustering granularity setting yields or shares the top performance score. (**b**) Box and whisker plot of the average performance scores (diamonds) attained over all superfamilies with a given method or setting. Note that the maximum observed value (top whisker) can be considerably higher than the others; these putative outlier values are indicated at the top.

The degree of sequence and (parent protein) function diversity varies highly among the domain superfamilies in Gene3D, corresponding to the different degrees of versatility and evolutionary 'success' of individual domain types [19]. Using function annotation data should therefore yield much better family partitionings than the use of clustering (at a fixed granularity level) alone. To confirm this expectation, FunFam families and GeMMA families were compared, based on a test set of 466 superfamilies with high-quality enzyme annotations (EC4s). The results are shown in Figure 5.

The bars in Figure 5a state how often the family partitionings obtained by using either the FunFam protocol or GeMMA clustering alone, with one of eleven fixed granularity settings, produce or share the top performance score (we used a fixed setting of E = 10^-40 ^in [19]). This relative measure does not take into account the absolute scores and how much they differ. The apparently significant advantage of the FunFam protocol is slightly diminished when looking at part b of the figure, a box and whisker plot showing the average performance scores obtained (lower is better; see above) and the distribution of the underlying raw scores. The data indicate that, in many cases, the E = 10^-10 ^clustering granularity setting yields a family partitioning that is close to the FunFam one in quality (AP scores of 1.85 vs. 1.54). On the other hand, the FunFam protocol performs significantly more consistently among the test superfamilies. This is indicated by the very short whiskers (indicating the minimum and maximum values observed) as compared with the remaining bars. Even when extreme outlier values (those close to the maximum values observed; see top of Figure 5b) are removed from the dataset, the general trend still holds (data not shown). Outliers may arise, for example, from superfamilies with incomplete EC annotation data.

### Protein function prediction performance

Using the FunFam domain families in the CAFA 2011 protein function prediction challenge, we performed among the top ten of 31 research groups taking part. The CAFA results further support the notion that our method generally outperforms a naïve BLAST [11] best-hit approach in terms of sensitivity; of course, only given that Gene3D domains can be assigned to a target protein in the first place. Notably, very few of the methods competing in CAFA explicitly used domain sequence or other domain-based data. The detailed CAFA results will be published elsewhere.

The CAFA team initially provided 48,298 potential target proteins (p-targets), of which 866 eventually became actual targets (a-targets), based on newly acquired experimental annotations. A total of 60,724 Gene3D domains could be assigned to 30,909 of the p-targets, that is, ~2 domains per assigned sequence on average. The rest of the target proteins were either not hit by any of the Gene3D superfamily models at all (12,752), or not well enough to justify a domain assignment (4,636). Taken together, these ~40% of domain-less p-targets therefore reflect largely unexplored parts of protein structure space, defining an upper boundary for our method's coverage. 633 (~75%) of all a-targets were assigned a total of 1,355 domains.

The minimum number of domains identified per p-target was 1, the maximum number was 93. The latter corresponds to target ID T43982 (COLA_DICDI), the >10,000 aa protein Colossin-A from slime mold. All its 93 structural domains are of the Immunoglobulin type (CATH 2.60.40.10). The minimum number of domain types (different CATH superfamilies) per p-target was 1, the maximum number was 12. The latter was found for a bifunctional glutamate/proline-tRNA-ligase protein from mouse, T29274 (SYEP_MOUSE). The a-target with the most (36) domains was T31967 (FAT_RAT), a rat protocadherin protein. A likely endocytic receptor from mouse, T29022 (SORL_MOUSE), exhibited the most (4) domain types among the a-targets.

The 60,724 identified p-target domains covered 1,662 different Gene3D superfamilies, of which 1,242 had an available FunFam library. 60,265 of the domain sequences fell into one of the latter superfamilies and hit at least one of the respective FunFam models. Only 459 sequences belonged to one of the remaining 420 superfamilies without a FunFam library. In other words, the library-less superfamilies do not significantly deteriorate the coverage of our method as a whole. This is because they represent very small, species-specific and largely unexplored parts of protein function space; notably, these superfamilies could be a good start to identify difficult targets for future assessments. Only three of the CAFA a-targets had domains exclusively belonging to superfamilies without a FunFam library.

After integrating the domain-based GO term predictions into whole-protein predictions, 30,672 of the 30,909 p-targets with Gene3D domains had at least one FunFam and GO term assigned. Again, the small difference indicates that the 420 library-less superfamilies did not impact our method's performance significantly. Accordingly, 630 of the 633 a-targets with identified domains could also be assigned to FunFams and, therefore, to GO terms.

## Discussion

The CAFA 2011 results put our domain-based approach among the top ten of 31 competing groups and 56 prediction methods. Apart from this quantitative result it would of course be interesting why and in which cases the domain-based approach outperforms such based on whole-protein sequences. A detailed analysis will yet have to be performed, a process in which the functional contribution of individual domains and domain families to the overall function (assignment) of specific target proteins must be manually reviewed. However, we feel that the following more general considerations are equally important.

### Family quality

Based on the results of the enzyme superfamily benchmark, it is clear that considering and processing the available high-quality GO annotations in the described FunFam protocol generally leads to higher functional purity and coherence of the produced families when compared to using any fixed level of sequence clustering granularity. Note that this is not a 'benchmark between equals' but rather, for the FunFam protocol, a measure of how well GO annotations, with their highly atomistic, incomplete and complex nature (see also below), can be used to establish sequence groups at a level of function conservation that is comparable to the EC4 level.

Compared to the EC system, our GO-based protocol has the benefit of much greater sequence coverage. On the downside, it is also much more complicated than it would have to be for EC annotations; there, sequences could simply be grouped by, for example, matching EC4.

### Protein function prediction performance

As seen by the detailed CAFA figures given above, our approach is currently not limited in coverage by superfamilies lacking annotation data and thus FunFams (functional knowledge) but by the considerable number of domain types that are known on the sequence level but have not yet been structurally characterised, that is, lack a CATH superfamily (structural knowledge). Notably, some of these sequences may nucleate novel superfamilies but have not yet entered or passed the CATH curation process.

In general, the protocol presented here should outperform whole-protein based methods in terms of prediction specificity and/or sensitivity in cases where a combination of domains is observed in a target protein that hasn't been observed before and, at the same time, the individual domains in the target protein fulfil those (partial protein) functions that are most often fulfilled by the other members of their FunFams (found in already characterised proteins). The latter is because the current, simple probabilistic protocol of family-to-function assignment will result in high-confidence function predictions (GO terms associated with high p-values) only where a certain function is associated with most of the family members. Of course, this is tied to the problem of family granularity: fine-grained families are functionally more conserved than coarse-grained ones, and it is therefore easier to achieve high p-values with them. However, their models may not always be sensitive enough to detect novel (outlier) member sequences in the first place. While we have tried to find a good compromise between these poles, radical improvements can only be expected when truly (manually curated) domain-specific annotations are available (see below).

### Domain function versus protein function

It must be noted that, in contrast to whole-protein (super)families, superfamilies of protein domains can be divided into functional families following two entirely different strategies. This is, based on (overall) protein function or (partial) domain function. If individual domains were annotated with specific functions, for example, 'binding' or 'catalytic activity', a protocol slightly different from the one presented here could be devised. This would not have to use the annotations of the parent proteins, in conjunction with a rather complicated heuristic protocol, but could identify functional families directly on the basis of the domain annotations. In the case of promiscuous domains, for example, the SH3 or PDZ interaction modules, this could lead to much larger domain families or even to superfamilies with a single family only. From a theoretical, evolutionary point of view (domains as functional building blocks) and for many practical purposes (for example, identifying conserved functional residues), such families, in character clearly different from whole-protein families, would be the more 'interesting' and useful kind of domain families. However, domain-specific function annotations in machine-readable format are thus far (as of 2011) only available in a relatively coarse form, in InterPro2GO; this may improve in the future.

With respect to GO, the example in Figure 4b can serve to illustrate the 'multi-domain' character of (some) GO terms. This is expected, given GO's aim to annotate whole proteins, however, it is a problem for domain-based protocols. P3 is such a term, describing the joint function of different domains. It is impossible in this case to establish a core set that reflects the function of domain I only (ATP hydrolysis), for two reasons. First, P2, despite its name 'hydrolase activity', which is a function of domain I only, is also a parent of P3. The child terms of P3, C1 and C2, therefore enter the core set. Second, even if that were not the case, these terms would still enter the core set via P1. This could only be avoided if the first protein was associated with more terms than just P1; for example, the missing 'hydrolase activity' for domain I. In this case, P1 would not be found in a sequence term set of minimum size (see above), unlike P2, and would therefore not play a role in identifying the core set.

Encouragingly, the practical application of the produced families in protein function prediction has shown that the current strategy of probabilistic family-to-function association, where different closely related functions and even functions mediated by co-occurring domains can be associated with a family, still leads to predictions with sufficient specificity.

### Functional grouping versus function prediction

Notably, optimising protein (domain) family identification and protein function prediction are two different goals. For prediction purposes, it is not required that all sequences with the same function(s) are pooled into a single family. Higher performance may even be reached when several families per homologous functional group exist; for example, one family for each domain of life or kingdom or, in the case of domains, one family per different parent protein domain architecture. In both cases, the combined detection sensitivity and the individual functional specificities of the family models can be increased when compared to producing a single family (model) only. This is because a wider and potentially uneven part of sequence space can be covered on the one hand (sensitivity) and, for example, kingdom-specific or domain-architecture specific aspects of protein function can be captured more selectively (specificity). Depending on the degree of diversity within the known and target sequence sets, the best results may even be achieved by using exhaustive pairwise sequence comparisons instead of models.

When trying to establish protein or domain families for uses other than ProFP only, for example, to provide a user-friendly means of browsing sequence, structure and function space, as desired for the CATH-Gene3D resource, a more balanced approach is required. To illustrate this further: it is not acceptable, both from a theoretic (biology) and a practical (usability) point-of-view, to produce thousands of small 'functional families' for a domain superfamily whose members come from proteins with only, for example, twenty different known and a few yet unknown functions. This is true regardless of whether or not a library of thousands of family models leads to higher performance in ProFP.

The outlined differences have gained even greater importance with the replacement of the EC system with the Gene Ontology as the standard means of function annotation: it is much more difficult to sensibly group sequences by their GO annotations than to merely compare the annotations of two individual sequences.

### Improving the family identification process

Currently, the initial core set of MF terms that is used to judge the functional coherence of domain sequence clusters in the family identification process is compiled based on the assumption that the parent proteins with the fewest function annotations are also those with the fewest domains. While this may be true in most cases, a safer approach should be used. This would require a simple protocol to identify all N-domain parent proteins for a given domain superfamily. For example, any protein with N domains, together covering at least 80% of its length, could be declared N-domain. For a given domain cluster, only the annotations stemming from those parent proteins with the smallest N would then be used to establish the initial core set.

### Improving the function prediction protocol

Family-based ProFP methods depend on a pre-established library of families and are therefore somewhat 'rigid'. In our particular case, this means that whenever a given target protein sequence does not have detectable Gene3D domains, or a detected domain represents an entirely novel part of its superfamily's sequence and/or function space (none of the FunFam models are hit), this leads to a total lack of predictions. In such cases, ProFP methods that are based on *ad-hoc *sequence comparisons can still provide functional hints (sensitivity advantage). These have to be backed up by multiple individual hits to putative (remote and partial) homologues to be reliable, though. This rationale is followed by methods such as Gotcha [30], PFP [31] and ESG [32]. The models used in family-based methods usually imply this reliability, given the underlying set of seed sequences is diverse enough (specificity advantage).

With the *de facto *standard PSI-BLAST [11] for sequence searches, also used by PFP and ESG, the traditional specificity/sensitivity dichotomy for sequence-based function prediction crumbles. The tool iterates between sequence comparison and profile generation, therefore trying to combine the advantages of *ad-hoc *search flexibility and profile sensitivity. Naturally, this comes at the price of losing some of the performance observed with either type of method in 'extreme' cases. The logical conclusion, given the ever-decreasing cost of computation, is that both family-based and *ad-hoc *methods should be run separately for ProFP, potentially adding the more balanced PSI-BLAST as a third 'vote'. Predicted functions would then be assigned higher probabilities the more methods or votes agree. Perhaps unsurprisingly, this strategy, at least the combination of BLAST and HMMER, was used in the best-performing methods in CAFA (to be published).

An improved ProFP workflow that includes domain information could look as follows. First, run the FunFam protocol discussed here. This can be augmented with scans against the InterPro domain family models and the use of the InterPro2GO mapping [7]. Coverage may so be increased, primarily because protein function space (as captured in InterPro member resources such as Pfam and PANTHER) is better explored than structure space (as captured in CATH). Second, scan all target proteins against a database of annotated sequences using PSI-BLAST; this step may additionally be repeated for each identified domain sequence. The BLAST results can be processed using published algorithms such as ESG [32]. Finally, after integration with the FunFam-based (and InterPro) predictions, output a list of function predictions with associated probabilities.

## Competing interests

The authors declare that they have no competing interests.

## Authors' contributions

RR conceived of and implemented the method. CAO helped to draft the manuscript. All authors read and approved the final manuscript.

## Declarations

This article has been published as part of *BMC Bioinformatics *Volume 14 Supplement 3, 2013: Proceedings of Automated Function Prediction SIG 2011 featuring the CAFA Challenge: Critical Assessment of Function Annotations. The full contents of the supplement are available online at http://www.biomedcentral.com/bmcbioinformatics/supplements/14/S3.
